# Interleukin 23 Produced by Hepatic Monocyte-Derived Macrophages Is Essential for the Development of Murine Primary Biliary Cholangitis

**DOI:** 10.3389/fimmu.2021.718841

**Published:** 2021-08-13

**Authors:** Debby Reuveni, Miriam R. Brezis, Eli Brazowski, Philip Vinestock, Patrick S. C. Leung, Paresh Thakker, M. Eric Gershwin, Ehud Zigmond

**Affiliations:** ^1^The Research Center for Digestive Tract and Liver Diseases, Tel Aviv Sourasky Medical Center, Tel Aviv, Israel; ^2^Sackler Faculty of Medicine, Tel Aviv University, Tel Aviv, Israel; ^3^Department of Pathology, Tel Aviv Sourasky Medical Center, Tel Aviv, Israel; ^4^Division of Rheumatology, Allergy and Clinical Immunology, University of California, Davis, Davis, CA, United States; ^5^Regeneron Pharmaceuticals, Inc., Tarrytown, NY, United States; ^6^Center for Autoimmune Liver Diseases, Tel Aviv Sourasky Medical Center, Tel Aviv, Israel

**Keywords:** primary biliary cholangitis, monocytes, macrophages, cytokines, interleukin-23

## Abstract

**Background and Aims:**

Primary Biliary Cholangitis (PBC) is an organ-specific autoimmune liver disease. Mononuclear phagocytes (MNPs), comprise of monocyte, dendritic cells and monocyte-derived macrophages, constitute major arm of the innate immune system known to be involved in the pathogenesis of autoimmune disorders. MNPs were shown to accumulate around intra-hepatic bile ducts in livers of PBC patients. Interleukin 23 (IL-23) is a pro-inflammatory cytokine. IL-23-positive cells were detected in livers of patients with advanced stage PBC and IL-23 serum levels found to be in correlation with PBC disease severity. Our overall goal was to assess the importance of IL-23 derived from MNPs in PBC pathogenesis.

**Methods:**

We utilized an inducible murine model of PBC and took advantage of transgenic mice targeting expression of IL-23 by specific MNP populations. Analysis included liver histology assessment, flow cytometry of hepatic immune cells and hepatic cytokine profile evaluation. Specific MNPs sub-populations were sorted and assessed for IL-23 expression levels.

**Results:**

Flow cytometry analysis of non-parenchymal liver cells in autoimmune cholangitis revealed massive infiltration of the liver by MNPs and neutrophils and a decrease in Kupffer cells numbers. In addition, a 4-fold increase in the incidence of hepatic IL-17A producing CD4^+^ T cells was found to be associated with an increase in hepatic IL23-p19 and IL17A expression levels. Disease severity was significantly ameliorated in both CD11c^cre^P19^flox/flox^ and CX_3_CR1^cre^P19 ^flox/flox^ mice as assessed by reduced portal inflammation and decreased hepatic expression of various inflammatory cytokines. Amelioration of disease severity was associated with reduction in IL-17A producing CD4^+^ T cells percentages and decreased hepatic IL23-p19 and IL17A expression levels. qRT-PCR analysis of sorted hepatic MNPs demonstrated high expression levels of IL-23 mRNA specifically by CX_3_CR1^hi^CD11c^+^ monocyte-derived macrophages.

**Conclusion:**

Our results indicate a major role for IL-23 produced by hepatic monocyte-derived macrophages in the pathogenesis of PBC. These results may pave the road for the development of new immune-based and cell specific therapeutic modalities for PBC patients not responding to current therapies.

## Introduction

Primary Biliary Cholangitis (PBC) is a chronic cholestatic liver disease characterized by progressive destruction of the intrahepatic bile ducts, leading to cholestasis, portal inflammation, fibrosis and potentially cirrhosis and liver failure (1). Mononuclear phagocytes (MNPs) are myeloid immune cells comprised of monocytes, macrophages (MFs) and dendritic cells (DCs). These cells are strategically positioned throughout the body tissues where they ingest and degrade dead cells, debris, and foreign material, and orchestrate inflammatory processes (2, 3). Studies in PBC patients, demonstrated accumulation of MNPs in the liver as well as impaired function of these cells. Mononuclear cells expressing the low-density lipoprotein binding glycoprotein CD68 were detected in the biliary epithelial layer of PBC patients, whereas in viral hepatitis these cells were scattered, and in normal livers were rarely seen around bile ducts (4). We have recently revealed a major role for Ly6C^hi^ monocytes in PBC pathogenesis and demonstrated significant amelioration of disease development by inhibiting the recruitment of these cells into the liver (5). Interleukin 23 (IL-23) is a pro-inflammatory cytokine belonging to the IL-12 family of heterodimeric cytokines (6). IL-12 and IL-23 share a common p40 chain as well as a common IL-12RB1 chain in their respective cognate receptors. IL-23 consists of two subunits, p19 and the shared p40 chain (6) and signals through a heterodimeric receptor consisting of the IL-23R chain and the shared IL- 12RB1 (6). IL-23 was shown to be essential for disease development in several models of autoimmune diseases, including psoriasis, inflammatory bowel disease and experimental autoimmune encephalomyelitis (7). The mechanism by which IL-23 exerts its pathogenic role has been mostly scrutinized in the context of Th17 cells, which were thought to mediate autoimmunity by secretion of IL-17 family cytokines. Studies in PBC patients showed higher IL-23p19 mRNA expression levels in PBMC’s from PBC patients that were correlated with PBC disease stages. Moreover, serum levels of IL-23 and IL-17 were positively correlated with serum GGT levels (8) and immunohistochemistry studies revealed expression of IL-23p19 in portal tracts of patients with advance disease (9). Mice deficient for IL-23 in all cells were found to be protected from PBC development (10), however the mechanisms involved and the cellular source of IL-23 has not been investigated. Of note, it has been suggested that IL-23 is expressed specifically by inflamed portal hepatocytes in PBC patients (9). Exposure to xenobiotics have been shown to be associated with break of immune tolerance in PBC (11). Xenobiotic modified PDC-E2 peptides mimic lipoic acid in a way that anti-PDC-E2 antibodies from PBC patients recognize them and results in higher titer reactivity than the native autoantigen. Notably, quantitative structure–activity relationship analysis identified 2-octynoic acid (2OA) as a xenobiotic candidate for antigenic modification of the PDC-E2 peptide (12–14). Thus, we have established a murine model for PBC based on immunization of mice with 2OA conjugated to bovine serum albumin (2OA-BSA) resulting in the appearance of anti-PDCE2 antibodies and histological lesions typical of autoimmune cholangitis 8 weeks following 2OA-BSA immunization (15).

By taking advantage of the 2OA-BSA inducible murine PBC model and transgenic mice that enabled targeting of IL-23 specifically in MNPs, we explored the importance of this cytokine expression uniquely by these cells, in the pathogenesis of autoimmune cholangitis.

## Material and Methods

### Mice

This study included the following animals: Cx3cr1-cre mice (JAX stock no. 025524, B6J.B6N(Cg)-Cx3cr1tm1.1(cre)Jung/J) (16), CD11c-cre mice (17), Il23-flox mice (18) and heterozygote Cx3cr1-gfp/+ reporter mice (19). Only female mice were used. Cre-negative littermates were used as controls. Animals were maintained in the animal facility of the Tel-Aviv Sourasky Medical Center. Mice had unlimited access to food and water, were kept in temperature and humidity-controlled rooms, and were maintained in a 12-h light/dark cycle. Use of animals was in accordance with the National Institutes of Health policy on the care and use of laboratory animals and was approved by the Tel-Aviv Sourasky Medical Center Animal Use and Care Committee.

### Preparation of Immunogen

2-octynoic acid (2OA) was purchased from Sigma-Aldrich (St Louis, MO, USA) and was conjugated with BSA (EMD Chemicals, Gibbstown, NJ, USA), as described previously (15). Briefly, 2OA was dissolved in dry dimethyl ether.

N-hydroxysuccinimide (NHS) was then added and the solution was cooled to 0°C and stirred for 20 minutes. Dicyclohexylcarbodimide was then added and the mixture was allowed to warm to ambient temperature overnight. The solution was filtered and concentrated. The product was then purified using flash chromatography (30% ethyl acetate/hexane). NHS-activated 2OA was dissolved in dimethyl sulphoxide and then coupled to the lysine residues of BSA. The solution was allowed to react for 3 hours followed by dialysis [phosphate-buffered saline (PBS)].

### Immunization

Female mice were immunized at 8 weeks of age by an intraperitoneally (i.p) injection of 2OA conjugated to BSA (2OA-BSA) at 1mg/ml per animal in the presence of complete Freund’s adjuvant (CFA) (Sigma-Aldrich) containing 10mg/ml of Mycobacterium tuberculosis strain H37Ra. Additionally, pertussis toxin (Sigma-Aldrich, 100ng/animal) was delivered i.p. on the day of immunization and 2 days after. A boost immunization was done 2 weeks following the initial immunization with 2OA-BSA in incomplete Freund’s adjuvant (Sigma-Aldrich). End of experiments were carried out 8 weeks following initial immunization.

### Histopathology

Mice livers were fixed in Formaldehyde 4% buffered (ph7.2), embedded in paraffin, cut into 4-μm sections, deparaffinized and stained with hematoxylin and eosin (H&E). A liver pathologist, blinded to treatment allocation, evaluated disease severity. 4 parameters were considered and each got a score between 0-2 (normal, moderate, or severe, respectively): 1.Portal infiltrate, 2.Bile duct damage and loss, 3. Granulomas formation and 4. Lobular inflammation, reaching a maximum score of 8.

### Isolation of Hepatic Non-Parenchymal Cells for Flow Cytometry

Hepatic non-parenchymal cells were isolated as previously described (20). In brief, mice were anesthetized and the livers were perfused with cold PBS. Cervical dislocation was performed and the livers were excised. Small fragments of liver were incubated (37°C, 250 rpm for 45minutes) in the presence of 5ml digestion buffer [5% FBS, 0.5 mg/ml collagenase IV (Sigma-Aldrich, Rehovot, Israel, C5138-500MG), 0.1 mg/ml Deoxyribonuclease I from bovine pancreas (Sigma-Aldrich, USA) in PBS^+/+^]. Following the incubation, the livers were filtered through 200μm wire mesh. To discard parenchymal cells, washings with PBS^-/-^ at 400 rpm, 4°C for 5 minutes was done three times harvesting the supernatant and discarding the parenchymal cell pellet. Last, supernatant was centrifuged at 1500rpm, 4°C, 5 minutes followed by ACK Lysing buffer (0.15 M NH4Cl, 0.01 M KHCO3) to exclude erythrocytes and washed with PBS^-/-^.

### Stimulation of T Cells for IL-17A Detection

Hepatic non-parenchymal cells were stimulated with 20 ng/ml PMA and 1µg/ml Ionomycin in the presence of 10µg/ml Brefeldin A in RPMI 1640 medium (supplemented with 10% heat-inactivated fetal bovine serum, 2 mM L-glutamine, 100 units ml^-^1 penicillin and 100 mg ml^-^1 streptomycin) at 37°C in a humidified incubator with 5% CO2.

Following 4h of stimulation, cells were washed and stained for the T cells surface markers TCRβ, CD3, CD4 and CD8. Then, cells were washed and fixed with the Flow Cytometry Fixation and Permeabilization Buffer Kit I (R&D Systems) for 30 min in 4°C. Cells were washed with permeabilization buffer and stained for intracellular IL-17A.

### Flow Cytometry Analysis and Sorting

Non-parenchymal liver cells were incubated with monoclonal antibody 2·4G2 for FcR blocking (BioLegend, San Diego, CA, USA) and then exposed at 4°C to a mixture of the following antibodies (dilutions are indicated):anti-mouse CD45 (clone 30-F11, 1:100), anti-mouse/human CD11b (clone M1/70, 1:300), anti-mouse Ly6C (clone HK1.4, 1:300), anti-mouse MHCII (clone M5/114.15.2, 1:200), anti-mouse CD11c (clone N418, 1:100), anti-mouse CD3ϵ (clone 145-2c11, 1:100), anti-mouse CD8a (clone 53-6.7, 1:100), anti-mouse CD4 (clone GK1.5, 1:100), anti-mouse TCRβ (clone 457-597, 1:100) all were purchased from BioLegend, San Diego, CA. Anti-mouse F4/80 (clone REA 126, 1:100) and anti-mouse Tim4 (clone REA999, 1:100) were purchased from Miltenyi Biotech.

For IL-17A staining, cells were first stained for surface markers, then the cells were fixed and permeabilized prior to intra-cellular staining with IL-17 (Clone TC11-18H10.1, 1:50, BioLegend). Cells were analyzed with BD FACS Canto™ II (BD Bioscience) or sorted with a FACSAria flow cytometer (BD Bioscience). Flow cytometry analysis was performed using FlowJo software (TreeStar, Ashland, OR).

### Quantitative Real-Time PCR

RNeasy Micro kit (QIAGEN) was used to isolate RNA from the liver. cDNA was prepared using the High-Capacity cDNA Reversed transcription kit (Applied Biosystems) according to the manufacturer’s instructions.

PCRs were performed with the SYBER green PCR Master Mix (Applied Biosystems) and with Taqman chemistry (Applied Biosystems) for IL-23p19 and IL-17A. Quantification was done with Step One software (V2.2). The Transcripts were tested, analyzed, and normalized relative to a housekeeping gene ribosomal protein, large P0 (RPLP0) and TATA-binding protein (TBP), for the genes IL-17A and IL-23p19 respectively.

### Statistical Analysis

The results are presented as mean ± SEM. Statistical analysis was performed using Two-tailed Student *t* test; *p* values < 0.05 were considered as significant.

## Results

### Monocyte and Monocyte-Derived Macrophages Become the Dominant Subsets of MNPs in the Liver Following Induction of Autoimmune Cholangitis

**Figure 1:**
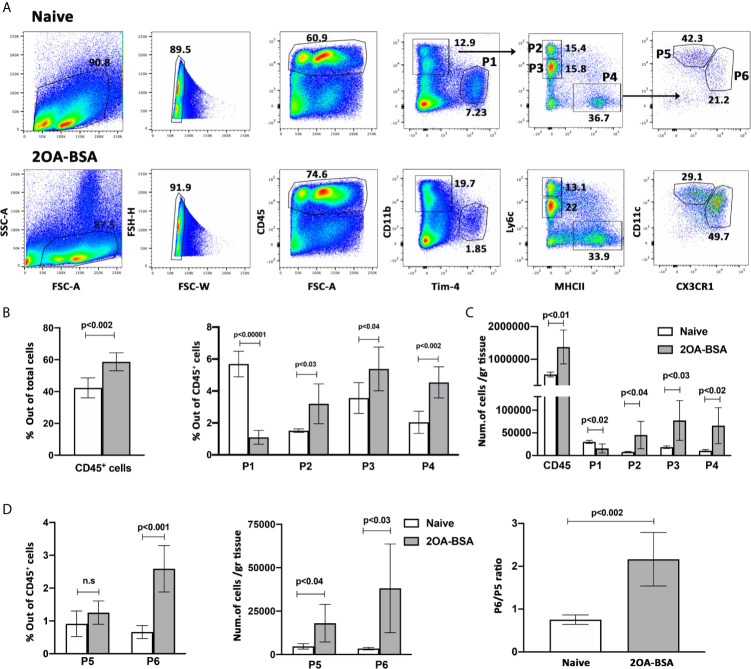
Monocyte and monocyte-derived macrophages become the dominant subset of MNPs in the liver following induction of autoimmune cholangitis. **(A)** Representative flow cytometry analyses of purified non-parenchymal liver cells from naïve mice (upper panel) and 2OA-BSA immunized mice 8 weeks following immunization (lower panel). **(B)** Graphical summary of flow cytometry analysis of non-parenchymal liver cells; left graph summarizes the percentages of CD45-positive cells out of total cells and the right graph summarizes the percentages of each cell population out of CD45-positive cells. **(C)** Graphical summary of cells numbers normalized for liver tissue mass for CD45-positive cells and P1-P4 cell subsets. **(D)** Graphical summary of flow cytometry analysis showing the percentages of P5 and P6 cell populations out of CD45-positive cells (left panel), absolute cell numbers of P5 and P6 cell populations normalized for liver tissue mass (middle panel), and P6/P5 ratio (right panel), in naïve mice vs. 2OA-BSA immunized mice 8 weeks post immunization. Results presented as mean ± SEM (n≥5) for each group. Results are representative of two independent experiments. *p* values < 0.05 were considered as significant (unpaired Student’s *t*-test).

The healthy liver harbors a large population of immune cells, however, under inflammatory conditions, the cellular composition of this hepatic immune compartment changes rapidly and dramatically (5, 20, 21). The hepatic mononuclear phagocytes (MNPs) comprise of monocyte, dendritic cells (DCs) and monocyte derived macrophages (MoMF). We have recently demonstrated that infiltration of Ly6C^hi^ monocytes to the liver is crucial to the pathogenesis of PBC and inhibition of the recruitment of these cells ameliorated all aspects of the disease (5). To examine the dynamics in the distribution of hepatic immune cells during autoimmune cholangitis, we performed multiparameter flow cytometry analyses on purified non-parenchymal liver cells from 2OA-BSA immunized mice. As shown in **Figure 1A** and **Supplementary Figure 1**, we have detected six major populations: Kupffer cells (P1) defined as CD11b^int^F4/80^hi^Tim-4^pos^, infiltrating classical monocytes (P2) defined as CD11b^pos^Ly6c^hi^MHC-II^neg^, Neutrophils (P3) defined as CD11b^hi^Ly6c^int^MHC-II^neg^, and another population we termed myeloid-APC (antigen presenting cells) (P4) defined as CD11b^pos^Ly6c^low^MHC-II^pos^Tim-4^neg^ CD11c^pos^CX_3_CR1^pos^ cells. Taking advantage of the Cx3cr1-gfp reporter mice (19), the P4 population can be further segregated into CD11c^hi^CX_3_CR1^int^ (P5) and CD11c^int^CX_3_CR1^hi^ (P6) sub-populations, that represent myeloid dendritic cells and monocyte-derived macrophages (MoMF), respectively. During murine autoimmune cholangitis, a significant increase in neutrophils, Ly6C^hi^ monocytes and myeloid-APCs population was found, whereas the KC population was significantly reduced (**Figures 1A, B**). Interestingly, focusing on myeloid-APCs (P4 population) under autoimmune cholangitis conditions, the CD11c^int^CX_3_CR1^hi^ MoMF became the dominant subset (**Figure 1C**).

### The IL-23-T_H_-17 Pathway Is Activated in the 2OA-BSA Murine PBC Model

To address the involvement of IL-23-T_H_-17 signaling pathway in experimental autoimmune cholangitis, we isolated non-parenchymal liver cells from naïve and 2OA-BSA immunized mice 8 weeks following immunization. Cells were stimulated with PMA and Ionomycin, as described in materials and methods, for detection of the intra-cellular cytokine IL-17A.

**Figure 2:**
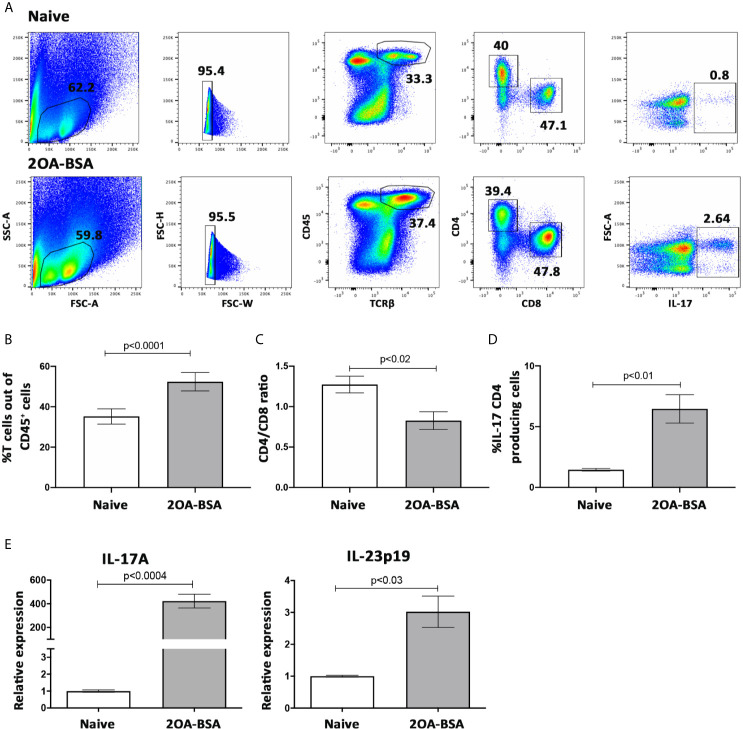
IL-23-T_H_-17 pathway is activated in the 2OA-BSA autoimmune cholangitis model. 2OA-BSA immunized mice and match-aged naïve mice were evaluated for: **(A)** Flow cytometry analyses of purified non-parenchymal liver cells, with a focus on intra hepatic T cells, from naïve mice (upper panel) and 2OA-BSA immunized mice (lower panel). **(B)** Graphical summary of the percentages of T cells out of CD45-positive cells. **(C)** Graphical summery of the CD4/CD8 T cell ratio. **(D)**. Graphical summery of the percentages of IL-17 CD4-positive producing cells. **(E)** Relative expression of hepatic IL-17A and IL-23p19 assessed by qPCR. Results presented as mean ± SEM (n≥5) for each group. Results are representative of two independent experiments. p values < 0.05 were considered as significant (unpaired Student’s t-test).

First, we looked at the T cell population in the liver and found that the percentage of T cells present in the liver following 2OA-BSA immunization were elevated and that the CD4/CD8 ratio was significantly decreased in the immunized mice, indicating massive infiltration of CD8 T cells (**Figures 2B, C**, respectively).

Flow cytometry analyses revealed a 4-fold increase in the incidence of hepatic IL-17A producing CD4^+^ T cells in autoimmune cholangitis (**Figures 2A, D**). Moreover, these results were accompanied by a 3-fold and a 300-fold increase in IL23-p19 and IL17A expression levels in autoimmune cholangitis versus healthy controls, respectively (**Figure 2E**), as evaluated in the whole liver tissue by qPCR.

### MNPs-Restricted IL-23 Deficient Mice Display Attenuated Disease Severity in the 2OA-BSA Autoimmune Cholangitis Model

**Figure 3:**
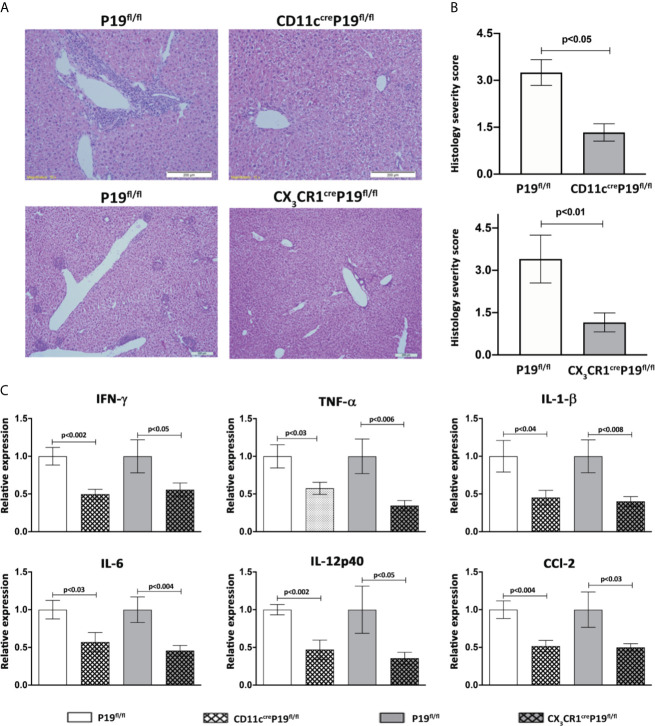
MNPs-restricted IL-23 deficient mice display attenuated disease severity in the 2OA-BSA autoimmune cholangitis model. MNPs-restricted IL-23 deficient and littermate controls mice were immunized with 2OA-BSA. Disease severity was evaluated 8 weeks following immunization by histological assessment of liver sections stained for hematoxylin &eosin, original magnification 40X **(A)**, accompanied by graphical summary of histological severity score **(B)**. Graphical summary depicting expression levels of hepatic cytokines of indicated mice **(C)**. All data presented as mean ± SEM (n≥10) for each group. Results are representative of two independent experiments. p values < 0.05 were considered as significant (unpaired Student’s t-test).

To evaluate the impact of MNPs restricted IL-23 deficiency on autoimmune cholangitis development we immunized CD11c^cre^P19^fl/fl^, CX_3_CR1^cre^P19^fl/fl^ and their P19^fl/fl^ littermates control mice with 2OA-BSA. A pronounced periportal infiltration of lymphocytes and mononuclear cells and bile ducts destruction was observed in P19^fl/fl^ mice, whereas, in both MNPs restricted IL-23 deficient mice almost no irregularities were detected (**Figure 3A**). Histology score given by a blinded pathologist revealed a significant attenuation in disease severity in both MNPs restricted IL-23 deficient mice (**Figure 3B**).

To further investigate the effect of MNPs restricted IL-23 deficiency on liver inflammation, expression of pro-inflammatory cytokines and chemokines in liver tissue were examined by quantitative real time PCR. The levels of *Ifn*γ**, *Tnfα*, *Il1β*, *Il6, Il12p40* and *Ccl2* were significantly increased in *P19^fl/fl^* mice but not in CD11c^cre^P19^fl/fl^ and/or CX_3_CR1^cre^P19^fl/fl^ mice immunized with 2OA-BSA. These results indicate that IL-23 derived from MNPs profoundly contributes to the development of pro-inflammatory response in experimental autoimmune cholangitis (**Figure 3C**).

### MNPs Restricted IL-23 Deficient Mice Display a Significant Reduction in the Frequency of Hepatic IL-17A Producing CD4^+^ T Cells and Diminished Activity of the IL-23-IL17 Axis in the Liver

**Figure 4:**
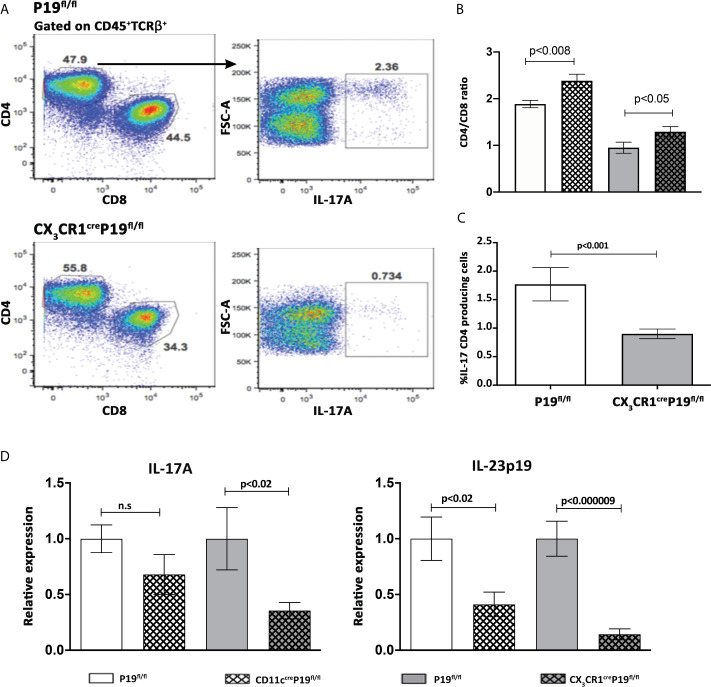
MNPs restricted IL-23 deficient mice display a significant diminished activity of the IL-23-IL17 axis in the liver. **(A)** Flow cytometry analyses of purified non-parenchymal liver cells from indicated groups of mice, 8 weeks following 2OA-BSA immunization. **(B)** Graphical summary of the percentages of CD4/CD8 T cells ratio from CD11c^cre^P19^fl/fl^ and CX_3_CR1^cre^P19^fl/fl^ immunized mice compared to their littermates’ controls. **(C)** Graphical summary of the percentages of IL-17 CD4^+^ producing cells in CX_3_CR1^cre^P19^fl/fl^ immunized mice vs. P19^fl/fl^ littermate controls. **(D)** Relative expression of hepatic IL-17A and IL-23p19 assessed by qPCR from indicated groups of mice, 8 weeks following 2OA-BSA immunization. Results presented as mean ± SEM (n≥10) for each group. Results are representative of two independent experiments p values < 0.05 were considered as significant (unpaired Student’s t-test).

We next attempted to determine the impact of specific MNPs-restricted IL-23 on the prevalence of hepatic IL-17A producing CD4^+^ T cells and the overall activity of IL-23 and IL-17 in the liver. Thus, 8 weeks following 2OA-BSA immunization, non-parenchymal cells were isolated from MNPs restricted IL-23 deficiency mice and from P19^fl/fl^ littermate controls and subjected to flow cytometry analysis. We have found a notable and significant elevation in the CD4/CD8 ratio in both MNPs restricted IL-23 deficient mice, implying reduced infiltration of CD8 T cells to the liver (**Figure 4B**). In the CX_3_CR1^cre^P19^fl/l^ mice, a significant reduction in hepatic IL-17A producing CD4^+^ T cells as compared to P19^fl/fl^ littermate controls was detected (**Figures 4A, C**). Moreover, qPCR analyses showed that the hepatic expression levels of IL-17A and IL-23p19 were significantly lower in CX_3_CR1^cre^P19^fl/l^ mice whereas only hepatic levels of IL-23p19 were decreased in CD11c^cre^P19^fl/fl^ (**Figure 4D**). These could be due to a more robust protection from autoimmune induced cholangitis in CX_3_CR1^cre^P19^fl/l^ mice, as was found also by liver histology assessment (**Figures 3A, B**).

### High Expression Levels of IL-23 mRNA by Hepatic MNPs Population Expressing Both CD11c and CX_3_CR1

**Figure 5:**
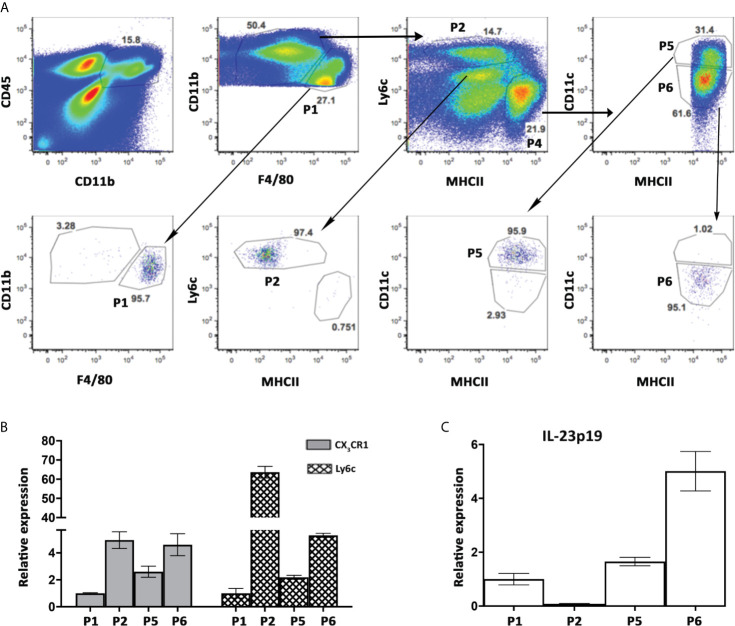
High expression levels of IL-23 mRNA by hepatic MNPs population expressing both CD11c and CX_3_CR1. **(A)** Flow cytometry sorting strategy of hepatic MNPs sub-populations from autoimmune cholangitis mice, 8 weeks following 2OA-BSA immunization (upper panel, and post-sorted cell populations (lower panel). **(B)** Graphical summary of qPCR analysis showing fold mRNA expression of CX_3_CR1, Ly6C **(B)** and IL-23p19 **(C)** in sorted cell populations. Graph depicts means (SEM) of two independent experiments; each experiment comprised of pooled RNA from 5 mice.

To specifically identify the subset of cells responsible for IL-23 secretion in the liver during autoimmune cholangitis, we sorted 4 populations of non-parenchymal cells from 2OA-BSA immunized mice: Kupffer cells (P1), Ly6C^hi^ Monocytes (P2) and the two subpopulations of myeloid APCs, P5 and P6 (see details in **Figures 1** and **5A**). This time, the sorting strategy to distinguish between myeloid DCs (P5) and MoMF (P6), was not based on CX_3_CR1 expression (as depicted in **Figure 1A**) rather by CD11c and MHCII. To validate our sorted populations, we performed qPCR analysis of sorted cells first for Ly6C and confirmed that it is specifically expressed by classical monocyte-P2 as expected. Increased Ly6C expression levels were detected also in P5 and P6 subsets, supporting their origin from infiltrating monocytes (**Figure 5B**). Next, we performed qPCR analysis of sorted cells for CX_3_CR1 expression levels and demonstrated higher levels in population P2 and P6, as expected from the flow cytometry analysis and strengthen P6 as the MoMF population (as described in **Figure 1A**). Of note, qPCR analysis of sorted cells from autoimmune cholangitis mice demonstrated unique high expression levels of IL-23 mRNA by P6 sub-population (**Figure 5C**), a CX_3_CR1^hi^ monocyte-derived macrophage that accumulate in the livers of animals with chronic autoimmune cholangitis (**Figure 1**).

## Discussion

In the current study we explored the importance of IL-23 expression by mononuclear phagocytes (MNPs) for the development of experimental autoimmune cholangitis. We showed that MNPs become the dominant cell subset in the liver during autoimmune cholangitis and that the IL-23-T_H_-17 pathway is activated. MNPs-restricted IL-23 deficient mice displayed attenuated disease severity, that was accompanied by a significant decrease in the percentage of hepatic IL-17 producing CD4 T cells. Sorting of hepatic APCs sub-populations revealed high expression levels of IL-23p19 mRNA specifically by CX_3_CR1^hi^ monocyte-derived macrophages, suggesting that IL-23 produced by these cells have a major role in the pathogenesis of PBC.

Interleukin 23 (IL-23) is a key pro-inflammatory cytokine important for the development of chronic inflammatory diseases. It is reported to be produced by various cellular sources including antigen-presenting cells as well as neutrophils, eosinophils and even non-immune cells (22–25). To specifically address the critical cell subset responsible for IL-23 production in experimental autoimmune cholangitis, we took advantage of two complementary conditional murine models. The CD11c^cre^ strain targets classical dendritic cells and plasmacytoid DCs, as well as monocyte-derived cells. However, expression of CD11c has been demonstrated in other lineages including NK cells, NKT cells, IgA+ plasma cells as well as some CD11c+ B and T cells (26–29). The Cx3cr1^cr^ *^e^* strain is more specific to monocytes-derived cells, however resident tissue macrophages (e.g., hepatic Kupffer cells) and subsets of mast cells and DCs were shown to be targeted as well (16, 30). Our results demonstrating decrease in disease severity in both CD11c^cre^IL-23p19^fl/fl^ and Cx3cr1^cre^IL-23p19^fl/f^ *^l^* mice, overcome this limitation of non-specific targeting and prove that MNPs are the critical cellular source of IL-23 in autoimmune cholangitis. It should be noted that although both strains showed amelioration of disease severity, a more robust improvement in disease severity accompanied by decrease in IL-23-IL17A signaling was found in Cx3cr1^cre^IL-23p19^fl/fl^ mice (**Figures 3**, **4**), implying monocyte derived macrophages as the major source of IL-23 in this pathology. This was corroborated by the remarkable elevated expression levels of IL-23 mRNA found in CX_3_CR1^hi^ monocyte-derived macrophages sorted from the livers of affected mice.

An important role of IL-23 in human PBC has been suggested as IL-23-positive cells were detected in livers of patients with advanced stage PBC and IL-23 serum levels were found to be in correlation with PBC disease severity (8, 9). Of note, Ustekinumab, an anti-IL-12/23 monoclonal antibody, has failed to achieve the alkaline phosphatase biochemistry endpoint in a phase 2 clinical trial (31). Nevertheless, modulation of the IL-12/23-related pathways was observed in a subset of patients with a decrease in alkaline phosphatase (31). Thus, assessment of other parameters, beside cholestatic liver enzymes is probably needed when evaluating response to biological treatments in PBC. Interestingly, in a transgenic murine model of PBC (dominant-negative form of transforming growth factor beta receptor type II) deletion of the p40 subunit, but not the p35 subunit resulted in amelioration of disease severity (32, 33), suggesting an important role for IL-23, but not IL-12 in this pathology. Moreover, it has been shown in other autoimmune disorders that IL-12 inhibition may even have a negative effect on disease course, a finding that has been attributed to a regulatory function of IL-12 *via* several mechanisms (34). Thus, specific IL-23 inhibition in a selected population of PBC patients with excessive inflammatory activity is worth an evaluation in a well-designed clinical trial.

In conclusion, we have recently revealed the critical role of hepatic monocyte infiltration for the development of experimental PBC; here, we have found that IL-23 production by these monocyte-derived cells drive this pathology. Our results support further evaluation of cell and cytokine specific therapeutic approaches in PBC patients not responding to current therapies.

## Data Availability Statement

The raw data supporting the conclusions of this article will be made available by the authors, without undue reservation.

## Ethics Statement

The animal study was reviewed and approved by Tel-Aviv Sourasky Medical Center Animal Use and Care Committee.

## Author Contributions

EZ, MG, and DR designed and guided the research. DR and MRB performed the animal experiments and analyzed most of the experiments. PV, PL, and PT contributed to research design and/or conducted experiments. EB performed the histological disease severity assessment. EZ and DR wrote the manuscript. All authors contributed to the article and approved the submitted version.

## Funding

This work was supported in part by the Israel Science Foundation (ISF) grant no. 2226/14, the United States–Israel Binational Science Foundation (BSF) grant no. 2013297, and by National Institutes of Health grant DK067003.

## Conflict of Interest

PT is employed by Regeneron Pharmaceuticals, Inc.

The remaining authors declare that the research was conducted in the absence of any commercial or financial relationships that could be construed as a potential conflict of interest.

## Publisher’s Note

All claims expressed in this article are solely those of the authors and do not necessarily represent those of their affiliated organizations, or those of the publisher, the editors and the reviewers. Any product that may be evaluated in this article, or claim that may be made by its manufacturer, is not guaranteed or endorsed by the publisher.

## Supplementary Material

Supplementary Figure 1 Kupffer cells are CD11b^int^Tim-4^pos^F4/80^hi.^ Flow cytometry strategy for KC identification by indicated Abs.

## Abbreviations

PBC, primary biliary cholangitis; 2OA-BSA, 2-octynoic acid conjugated to bovine serum albumin; CX_3_CR1, C-X3-C motif chemokine receptor 1; DC, dendritic cells; MoMF, monocyte-derived macrophages H&E, hematoxylin and eosin; IFN-γ, interferon gamma; IL-1β, interleukin 1 beta; IL-17, interleukin 17, IL-12, interleukin 12; IL-23, interleukin 23; Ccl2, chemokine (C-C motif) ligand 2; TNF-α, tumor necrosis factor alpha.
