# Supplementary figures and images for: Interleukin 23 Produced by Hepatic Monocyte-Derived Macrophages Is Essential for the Development of Murine Primary Biliary Cholangitis
Source: Front Immunol. 2021 Aug 13;12:718841. doi: 10.3389/fimmu.2021.718841 (PMC8414574; doi:10.3389/fimmu.2021.718841)

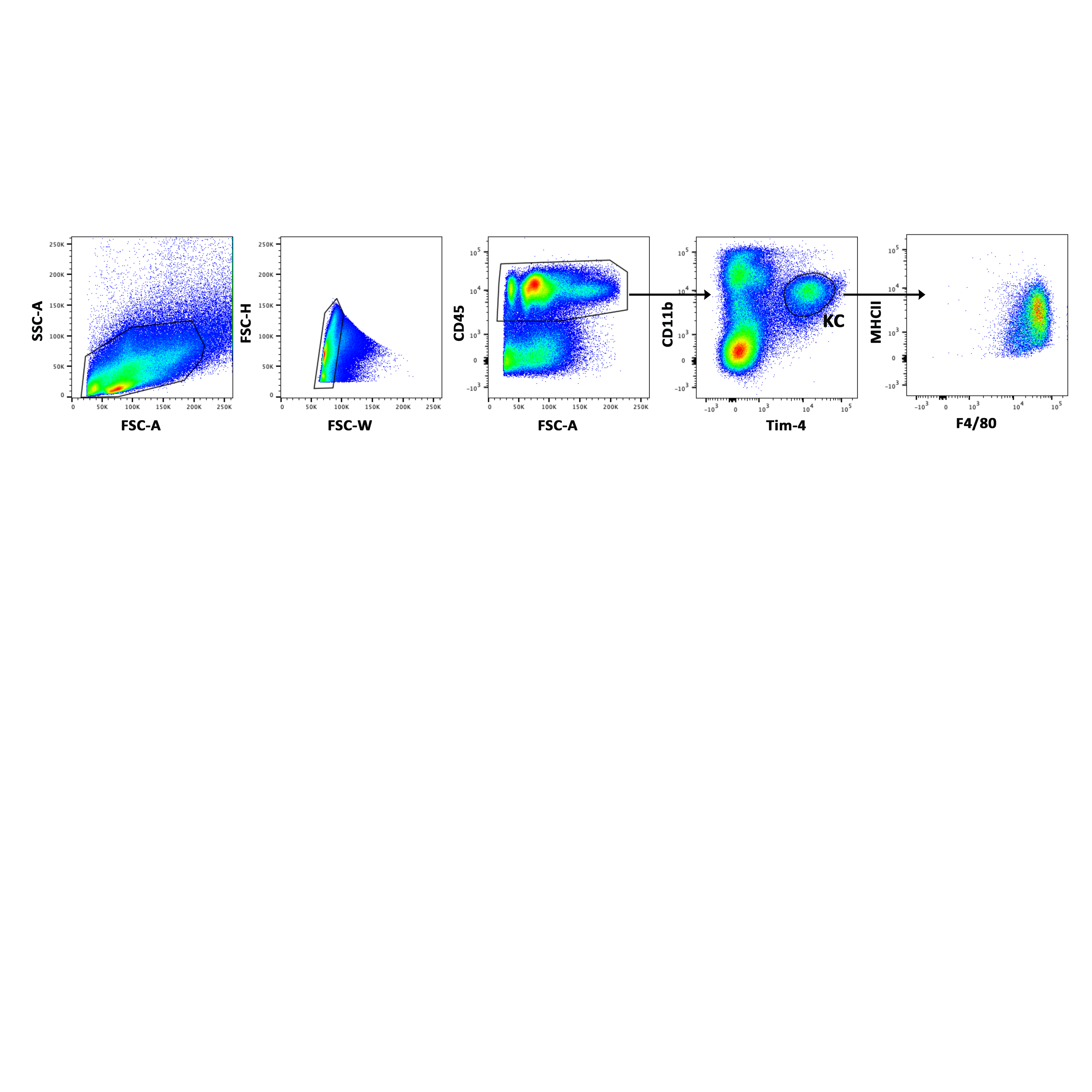

Supplement: Supplementary Figure 1 — Kupffer cells are CD11bintTim-4posF4/80hi. Flow cytometry strategy for KC identification by indicated Abs. [file Image_1.jpeg]
